# Strategies for improved temporal response of glass-based optical switches

**DOI:** 10.1038/s41598-021-04218-3

**Published:** 2022-01-07

**Authors:** Matteo Calvarese, Petra Paiè, Francesco Ceccarelli, Federico Sala, Andrea Bassi, Roberto Osellame, Francesca Bragheri

**Affiliations:** 1 Istituto di Fotonica e Nanotecnologie (IFN)-CNR, Piazza Leonardo da Vinci 32, 20133 Milan, Italy; 2 Dipartimento di Fisica-Politecnico di Milano, Piazza Leonardo da Vinci 32, 20133 Milan, Italy

**Keywords:** Integrated optics, Photonic devices

## Abstract

We present an optimization of the dynamics of integrated optical switches based on thermal phase shifters. These devices have been fabricated in the volume of glass substrates by femtosecond laser micromachining and are constituted by an integrated Mach–Zehnder interferometer and a superficial heater. Simulations, surface micromachining and innovative layouts allowed us to improve the temporal response of the optical switches down to a few milliseconds. In addition, taking advantage of an electrical pulse shaping approach where an optimized voltage signal is applied to the heater, we proved a switching time as low as 78 µs, about two orders of magnitude shorter with respect to the current state of the art of thermally-actuated optical switches in glass.

## Introduction

Circuit stability, compactness as well as precise alignment between the components make integrated optics an appealing approach for a wide set of applications. These include optical communication, sensing, metrology, quantum optics and even lab-on-a-chip devices^1^. The capability of performing circuit reconfigurability is of paramount relevance for many of the above-mentioned applications^2,3^. Different approaches can be implemented to achieve this feature, which can be based on refraction or absorption processes, whether the real or the imaginary part of refractive index is modified. The material of the substrate, together with the used fabrication technique determine the properties of the circuit and the optimal modulation approach. Several technologies and substrates are currently used for integrated optics applications, including silicon on insulator, silicon nitride, indium phosphide, lithium niobate and glasses. Kerr or Pockels effects can be efficiently used if the substrate presents high nonlinear or electro-optic coefficients (as in the case of lithium niobate^4^). Alternatively, plasma dispersion effect is commonly used in silicon photonics^5^, in which free carrier concentration modification, alters both the real and the imaginary part of refractive index, therefore, affecting the propagation losses. Both methods allow megahertz or gigahertz modulation ranges. Reconfigurability can also be achieved by thermo-optic effect, exploiting the temperature dependence of the refractive index^6–9^. This approach is characterized by the implementation simplicity, the stability and accuracy in light modulation together with the lack of losses increase during the heating. These properties, combined with the good thermo-optic coefficient of certain substrates (i.e., glass and silicon), make this method competitive with respect to other techniques, despite the lower achievable modulation frequencies. To obtain integrated optical circuits, femtosecond laser micromachining (FLM) is a valuable alternative to standard lithographic approaches^10,11^. This technique is based on non-linear absorption processes that occur when focusing a femtosecond laser beam on transparent substrates, such as glasses. This leads to a material modification whose properties depend on the irradiation parameters. Among the possible structural changes, a localized refractive index increase with respect to the pristine material can be induced, which enables direct waveguide writing. This is a maskless approach, therefore characterized by fast prototyping. The fabricated integrated optical circuits are characterized by low losses, three-dimensional layouts and a straightforward coupling with optical fibers, due to the good modal overlap^12,13^. In addition, FLM allows fabricating both integrated optics and microfluidic channels, which can be used in synergy for the realization of advanced lab-on-a-chip devices in fused silica and borosilicate glasses^14,15^. All these reasons make FLM an appealing alternative to lithographic techniques. In recent years, several works in literature have presented thermo-optical modulators based on integrated optical circuits in glass substrates fabricated by FLM. Chaboyer et al.^16^ demonstrated multi-arm quantum interferometry in a 40 mm long glass chip with a 3D interferometer in which the arms present different distances with respect to the heater placed on the surface. Flamini et al.^17^ reported a reconfigurable integrated photonic circuit for quantum information at telecom wavelengths, where FLM has been used to fabricate the integrated optical circuit, as well as to define the layout of a superficial heater after ubiquitous gold deposition. In subsequent works, laser micromachining has been successfully implemented to structure the substrate and consequently to reduce the power dissipation in the modulators. Chaboyer et al.^18^ have inscribed superficial trenches between the two arms of the Mach–Zehnder interferometer (MZI) to increase thermal isolation. Demonstrating a power dissipation for a 2π phase shift of about 200 mW, they have improved the phase tuning efficiency by a factor of two. Ceccarelli and colleagues^19^ have further pushed the limits of this approach by 3D structuring the photonic circuit and proving a power dissipation for a 2π phase shift of only 37 mW and 1 mW when performing the measurements in air and in vacuum, respectively. This is a fundamental result that demonstrates the possibility of device scalability while keeping a low total dissipated power. Nevertheless, a major limitation of these circuits is still given by the slow dynamic responses that are characterized by switching times of the order of tens or hundreds of milliseconds^20,21^. Despite these values are compatible with many applications, where a reconfigurable but static operation is needed, they can constitute a major limitation when fast and frequent device reconfiguration is required. In this work we focus on improving the dynamic response of modulators based on the thermo-optic effect in glass photonic circuits fabricated by FLM. We have optimized the device layout and investigated the effects of trenches, validating our simulations with experimental measurements. In addition, by combining these results with a pulse shaping approach we have optimized the applied voltage function and proven a switching time of more than two orders of magnitude shorter than modulators previously fabricated in glass substrates.

## Design, methods and material

### Design

**Figure 1:**
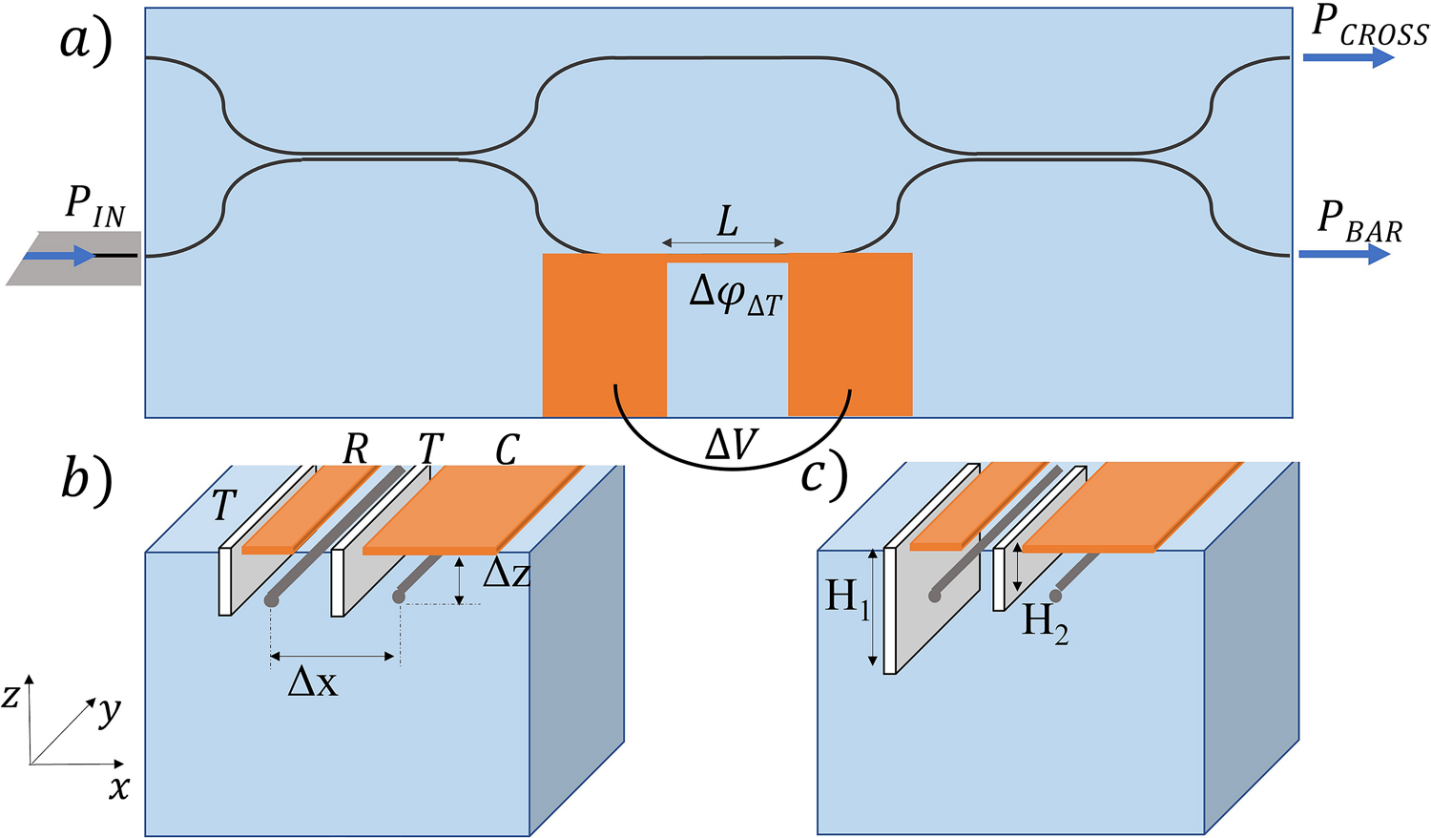
(**a**) Schematic of the MZI device in the *standard configuration*. (**b**) and (**c**) Two *advanced configurations* introduced to speed up the dynamic response of the optical modulator. R, C and T stands for resistor, conductive layer, and trench, respectively. Δz and Δx correspond to the depth of the waveguides from the surface and the reciprocal distance. H1 and H2 indicate the height of the trenches, which is the same for the two trenches in panel b (*symmetric configuration*) and different in panel c (*asymmetric configuration*).

The thermo-optic effect can be used in combination with a Mach–Zehnder Interferometer (MZI) to obtain an optical switch. The transfer matrix of the MZI depends on the phase difference between the two central arms. If we consider the case of an ideal MZI, with balanced splitting ratio along the two integrated couplers, negligible losses and with light coupled in one of the two inputs, we obtain in correspondence of the two outputs (bar and cross, as in Fig. 1a) the following power distribution:

1 $$\begin{array}{*{20}c} {P_{bar} = P_{IN} \sin^{2} \left( {\frac{\Delta \phi }{2}} \right) } \\ \end{array}$$

2 $$\begin{array}{*{20}c} {P_{cross} = P_{IN} \cos^{2} \left( {\frac{\Delta \phi }{2}} \right) } \\ \end{array}$$

where $P_{IN}$ is the optical power coupled at the input and $\Delta \phi$ is the optical phase difference between the two arms. Therefore, by controlling the phase difference it is possible to switch the power from one output to the other. Figure 1a illustrates the schematic design of our device, where a heater (i.e. a resistor) is fabricated above one of the two arms of the MZI. Applying a controlled voltage over the resistor induces heating of the substrate; the temperature variation is proportional to the dissipated power. Due to the asymmetric position of the resistor with respect to the optical circuit, the two arms of the MZI reach a different temperature. This, in turns, gives rise to a difference in refractive index. The temperature dependence of the refractive index for a wide range of values can be approximated as linear:

3 $$\begin{array}{*{20}c} {n\left( T \right) = n\left( {T_{0} } \right) + \alpha \left( {T - T_{0} } \right)} \\ \end{array}$$

where $\alpha = dn/dT$ is the thermo-optic coefficient of the substrate, and $n\left( {T_{0} } \right)$ is the refractive index at room temperature $\left( {T_{0} } \right)$. The heating generates a temperature dependent phase difference Δ*ϕ*_ΔT_ among the two arms of the MZI, as follows:

4 $$\begin{array}{*{20}c} {\Delta \phi_{\Delta T} = \frac{2\pi }{\lambda }\alpha L\Delta T } \\ \end{array}$$

where *L* is the resistor length, and *λ* the wavelength guided in the MZI. Therefore, by applying a certain voltage over the resistor, it is possible to control the light distribution at the MZI output, switching between the two ports. To optimize the dynamic response, we have focused first in optimizing the layout of the device in its *standard configuration* (Fig. 1a). This has been done by optimizing the values of Δz and Δx, which are the depth in the substrate at which the waveguides are irradiated and the distance among the two arms, respectively. Subsequently, we have explored *advanced configurations*, characterized by the presence of a conductive layer above the second waveguide and superficial trenches at the resistor’s sides (Fig. 1b, c).

### Device fabrication and experimental setup

The device fabrication is composed by different steps. First, we have fabricated the integrated optical circuit by FLM in borosilicate glass (Eagle XG, Corning). Subsequently, following a ubiquitous gold nanolayer deposition, we have selectively removed the metal by femtosecond laser ablation, to define the resistors pattern.

For all FLM tasks we have used a Yb:KYW cavity-dumped mode-locked laser oscillator, with pulses at a wavelength of 1030 nm, a duration of 300 fs, and a repetition rate of 1 MHz. A first optimization has allowed us identifying the best parameters to obtain single mode waveguides at 532 nm. We have used a 50x, 0.65 NA as focusing objective, an average laser power of 230 mW, a scan velocity of 40 mm/s and a multiscan approach based on 12 overlapping irradiations. The waveguides have been irradiated at a distance of 15 µm from the bottom surface of the glass substrate. Following a previously optimized annealing process up to 750 °C^22^, we have obtained single mode waveguides with propagation losses of about 0.3 dB/cm and symmetric mode dimensions of about 2.7 × 2.8 μm^2^ at a wavelength of 532 nm. We have investigated the integrated couplers to obtain a symmetric splitting ratio between the two arms. Indeed, we have fabricated and analyzed the output splitting ratio of 15 couplers with the same distance between the waveguides in the coupling region (5 μm) and with an increasing length of interaction (from 0 to 3 mm). From our experimental characterization we have estimated an interaction length of 0.75 mm to have a symmetric power splitting.

Regarding the ubiquitous metal deposition process, first 2 nm of chromium have been deposited over the substrate to enhance gold to glass adhesion^21,23^. Subsequently, 80 nm of gold have been deposited using a magnetron sputtering system. A thermal annealing process up to 500 °C has been introduced to favor the resistors stability over time.

The ablation for the resistor layout is carried out by using the above mentioned femtosecond laser, a 10x, 0.25 NA focusing objective, 200 mW of average laser power and 2 mm/s scan velocity. These parameters have been selected as they allow to remove the metal without affecting the surface quality of the glass underneath. The resistor is 3 mm long and 15 μm large, which corresponds to a resistance value of about 115 Ω. For the fabrication of superficial trenches multiple lines have been irradiated. In particular, 5 overlapping scans have been repeated at different depths from the surface, covering the entire height of the trench with an inner separation of 2 μm along the vertical axis (z axis in Fig. 1). Additional irradiation parameters are 800 mW of average laser power and 0.2 mm/s scan velocity.

### Simulations

**Figure 2:**
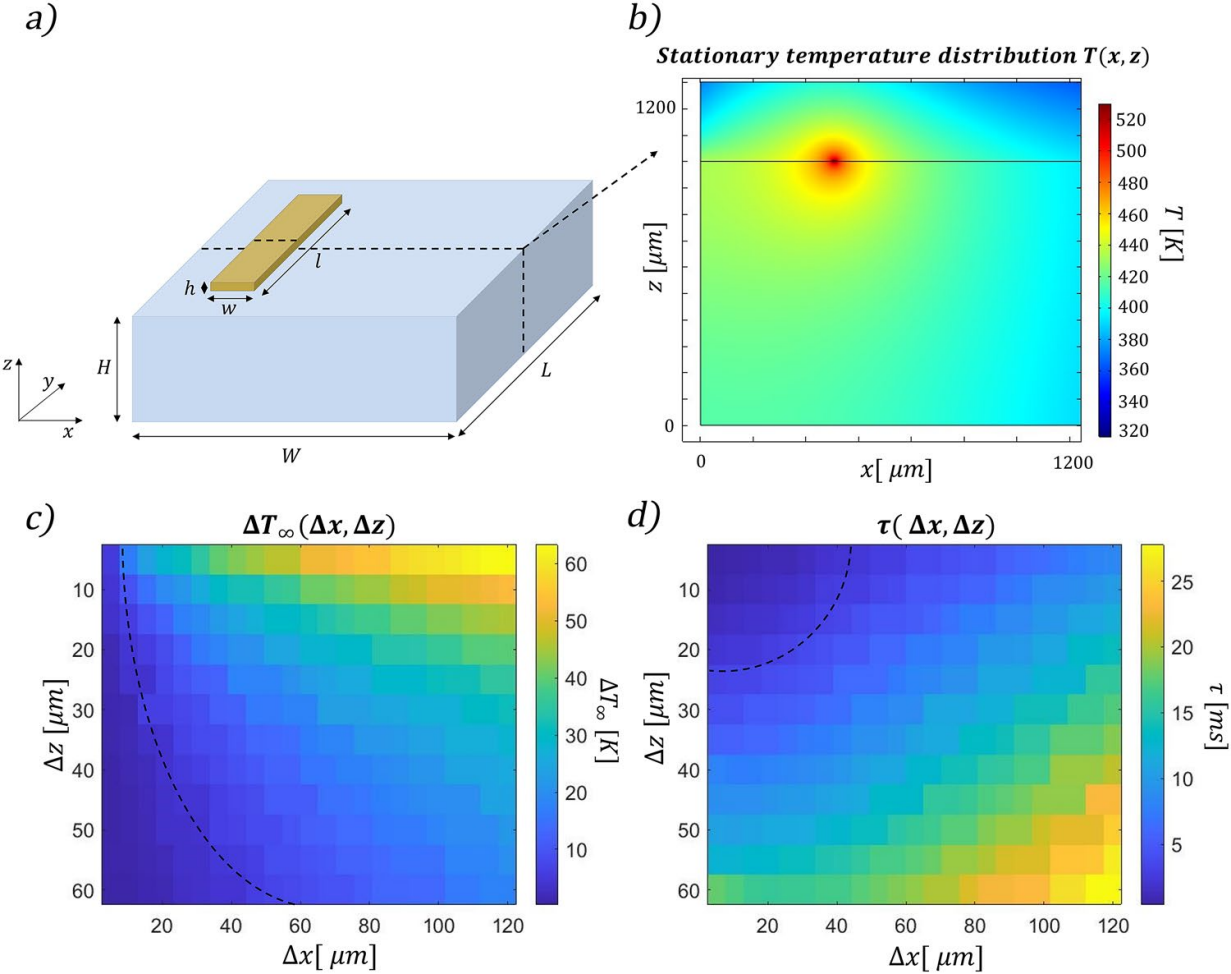
(**a**) Layout of the device simulated in COMSOL Multiphysics and (**b**) temperature distribution in the device cross-section. Panels (**c**) and (**d**) illustrate the final difference in temperature among the two arms of the MZI (ΔT_*∞*_) and the time (τ) required to reach the 90% of the asymptotic value, depending on the position of the waveguides (Δz and Δx). Dashed lines indicate threshold values for ΔT_*∞*_ and τ.

COMSOL Multiphysics has been used to simulate both the static and dynamic response of the devices to an applied power step. The *Heat transfer module* allowed to investigate the temperature distribution in the substrate in correspondence of a certain dissipated power. To model the device, three different domains have been introduced. A block of borosilicate glass with dimensions of 5 mm × 5 mm × 1 mm (L × W × H in Fig. 2a), a thin layer of gold of 3 mm × 15 μm × 1 μm (l × w × h in Fig. 2a), set as heat source with an emitting power P and a layer of air 5 mm x 5 mm × 0.3 mm above the others to consider the effect of heat dissipation by conduction in air. The physical parameters of the materials used have been imported from COMSOL libraries. A physics-controlled mesh has been used to adapt the voxel size to the dimensions of the features along the structure. The thickness of the gold layer considered in the simulations overestimates the one that we have experimentally used. Nevertheless, a thinner one would have required a much longer computational time. In addition, we expect that this choice does not affect too much the reliability of our simulations, considering that three sides of the resistor are in contact with air, which has a significantly lower thermal conductivity with respect to glass. Therefore, all the power is dissipated through the glass substrate.

## Results and discussion

### Simulations

**Figure 3:**
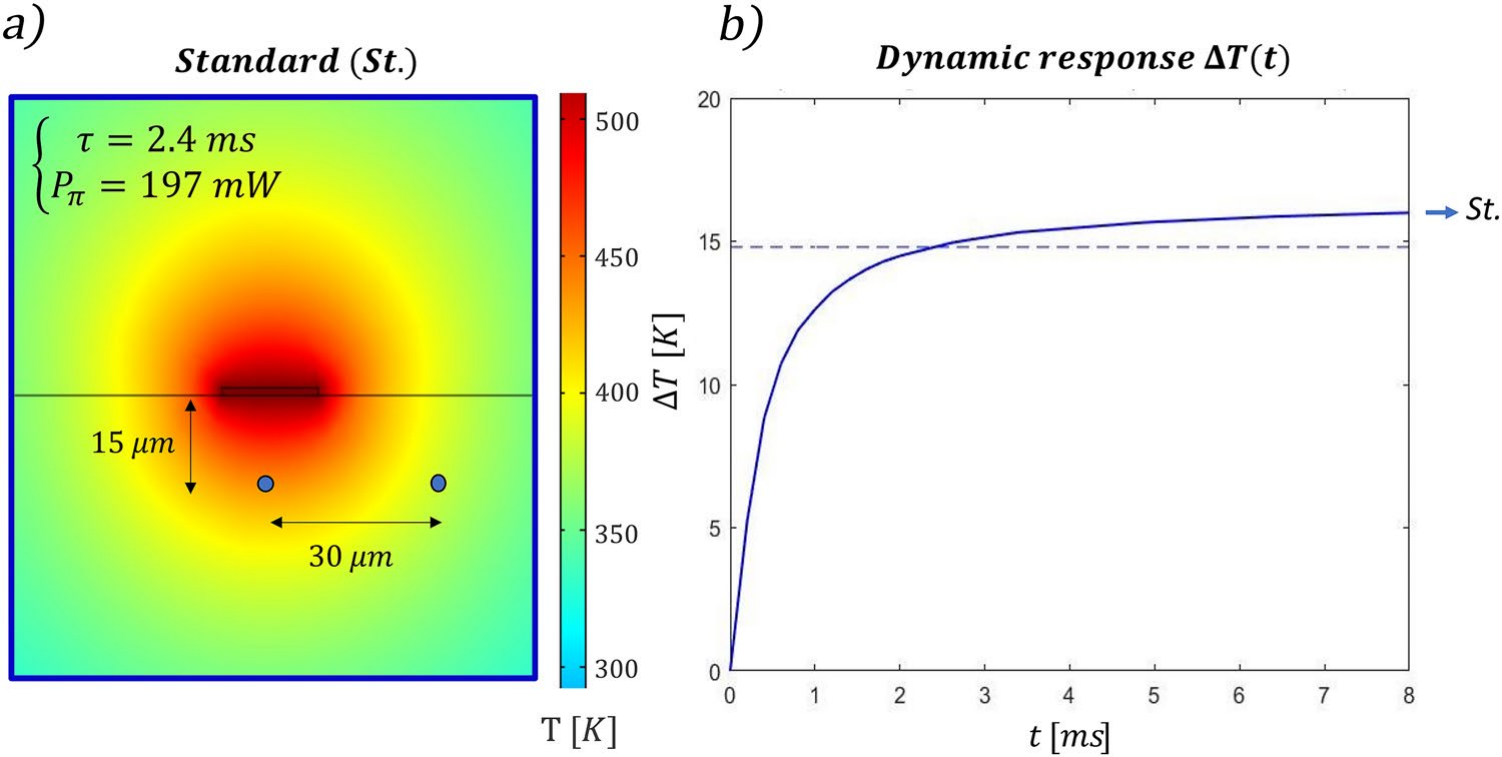
(**a**) Temperature distribution in the device cross-section when 250 mW are applied to the resistor. (**b**) Dynamic response of the device under the same dissipated power, the 90% of the steady state condition is reached after about 2.4 ms.

**Figure 4:**
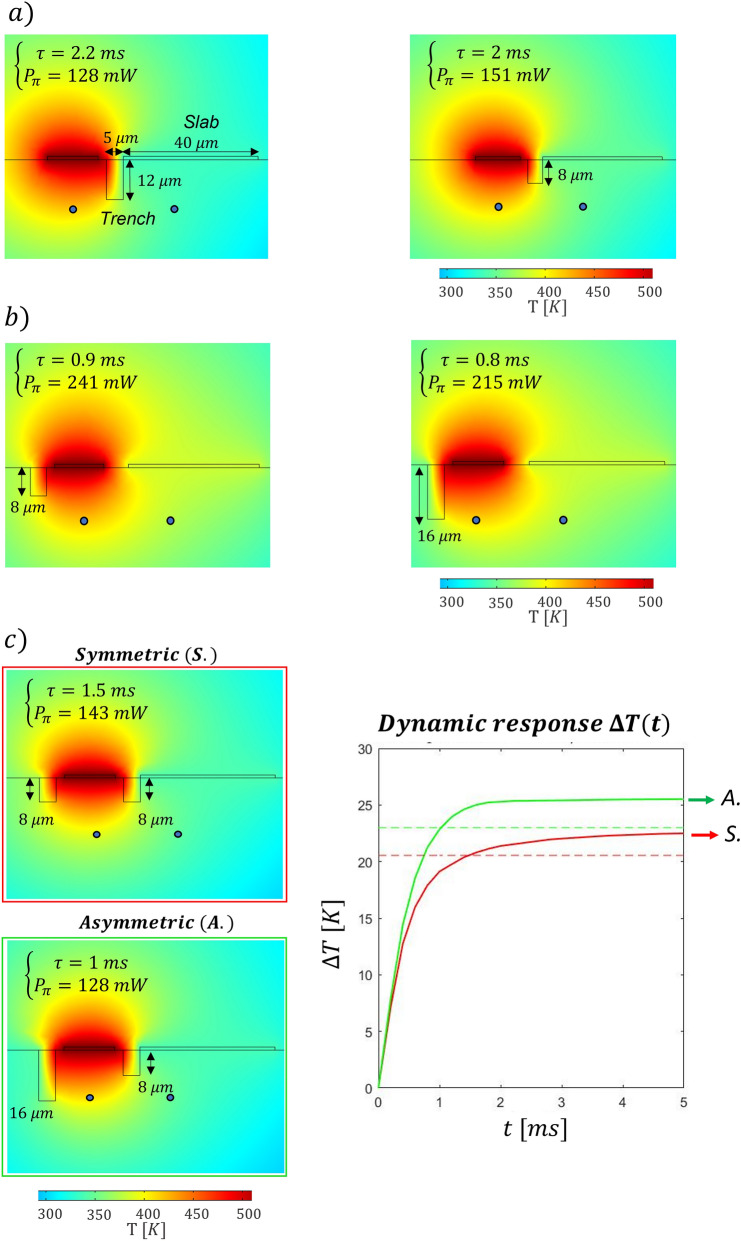
Simulated temperature distribution in different configurations under a power dissipation of 250 mW. Effect of the right-side (**a**) and left-side (**b**) trench on power consumption and system dynamics. Comparison of the symmetric (S.) and asymmetric (A.) configuration under a dissipated power of 250 mW (**c**).

Using COMSOL Multiphysics, we have first investigated the effects of the layout of what we refer to as *standard configuration*, which is constituted by the integrated MZI and the superficial resistor (Fig. 1a). We have first retrieved the steady state temperature distribution in the substrate’s cross section, with a dissipated power *P* = 250 mW (Fig. 2a, b). As expected, we have noted that closer to the resistor the temperature is higher. In addition, we have observed that the difference in temperature among two points reaches its steady state value faster when these points are closer to the resistors, as it will be thoroughly discussed later in this paragraph. Considering both these aspects, we have decided to place one of the two waveguides directly below the resistor (at a depth Δz), symmetrically placed in its center. The second waveguide, instead, is located at the same depth into the substrate, but shifted of a certain Δx. To retrieve the optimal values of Δz and Δx, we have first investigated the difference in temperature reached by the two waveguides in the steady state condition (ΔT_∞_) with 250 mW of dissipated power. The higher this value, the lower the power required to induce the π-shift necessary to perform the switch (see Eq. 4). Figure 2c is a 2D map that illustrates ΔT_∞_ reached between the two arms, depending on the position of the waveguides (Δz and Δx). To maximize this value, the two waveguides should be placed close to the surface and distant from each other. The black dashed line is an isothermal line whose points correspond to different combination of waveguides’ position that present a ΔT_∞_ of 13 °C, the necessary value to induce a π-phase difference at 532 nm. All the points above that line reach a higher difference of temperature and permit a π-shift with less power. Secondly, we have investigated the dynamic response analyzing the time τ required to reach the 90% of the steady state value. In the map of Fig. 2d different values of τ are reported in correspondence of different combinations of waveguides’ position in the substrate cross-section. In this case a threshold value of 5 ms is indicated by the black line. We have therefore combined the two conditions, to retrieve the range of acceptable configurations. Both the analyses suggest minimizing Δz, while they lead to opposite conclusions regarding the distance between the waveguides. Indeed, at a fixed Δz, it is important to enhance Δx to maximize the difference of temperature reached (ΔT_∞_). Conversely, to speed up the dynamics, the two arms should be as close as possible, thus allowing to reach the steady state condition faster. We have chosen to fabricate the integrated MZI at the shallowest possible depth that does not cause surface ablation when writing the waveguides. In our case, this depth corresponds to 15 µm from the surface. As shown by the simulations reported in Fig. 2d, the shallower the waveguide the faster the switching dynamics, therefore further improvement in the time response can be expected by developing waveguide writing closer to the surface while avoiding ablation. At the chosen depth, considering that the range of Δx allowed by the two thresholds spans from 23 to 43 µm, we have selected a distance between the waveguides of 30 µm. In this configuration the power required to obtain a π shift is 197 mW and τ is about 2.4 ms, as illustrated in Fig. 3. Subsequently, we have explored the possibility of a further optimization of the device with *advanced configurations*, introducing both trenches and a conductive slab of gold. Recently, Ceccarelli et al.^18^ proved that deep bridge-like trenches can thermically isolate one of the two arms, decreasing the power needed for the shift. Nevertheless, this has negatively affected the device dynamics, with a switching time that is about three times higher with respect to the standard approach with no trenches. Here, starting from the previously optimized standard configuration, we have investigated new layouts of trenches that do not entirely isolate one of the two waveguides from the substrate, as proposed by Ceccarelli et al.^18^, with the aim of finding a possible trade-off between temporal response and power dissipation. In particular, we have tried to optimize the switching time, maintaining the power dissipation below 250 mW. First, we have introduced a metallic slab on the glass surface, which favors heat diffusion and permits reaching the steady-state temperature difference in a shorter time, but at the cost of increasing the power consumption. In addition, we have analyzed the effect of trenches size and position as summarized in Fig. 4. Figure 4a illustrates how the presence of the trench on the right side of the electrode modifies the system, comparing two layouts that differ only for the trench depths. Its presence affects mainly the power dissipation, but it slightly improves also the dynamic response; the shallow trench is the one with the highest impact on the system dynamics. Figure 4b investigates the effects of the trench on the left side of the electrode. In this case the deepest trench is the optimal one for both power dissipation and system dynamics. Following this analysis, we have studied two different devices, which are characterized by superficial trenches with different dimensions and the same metallic slab (a nanolayer of gold 40 µm wide). A first layout, named s*ymmetric,* is characterized by two superficial trenches that are 5 µm wide and 8 µm deep. A second one, named a*symmetric,* presents two different trenches, one with the same dimensions as in the s*ymmetric* case, and the other one with dimensions twice as deep. Figure 4.c illustrates the steady state temperature spatial distribution in these configurations and it compares the simulation of the two dynamic responses when 250mW are dissipated in the resistor. It is interesting to note the following aspects: (1) both devices present a faster dynamic response with respect to the s*tandard* configuration illustrated in Fig. 3b. (2) The a*symmetric* configuration is the fastest, as it reaches the 90% of its regime value in about 1.0 ms, while 1.5 ms is required in the s*ymmetric* device case. This indicates that the external trench height also affects the dynamic response of the device. (3) Both devices reach a higher temperature difference with respect to the *standard* configuration (whose ΔT_∞_ is about 17 K). (4) The *asymmetric* configuration reaches the highest temperature difference. This implies that the power required to perform a π-shift (P_π_) is also lower with respect to the other two configurations. In fact, the simulated P_π_ corresponds to 128 mW, 143 mW and 197 mW for the *asymmetric*, *symmetric* and *standard* configurations, respectively.

### Experimental validation

**Figure 5:**
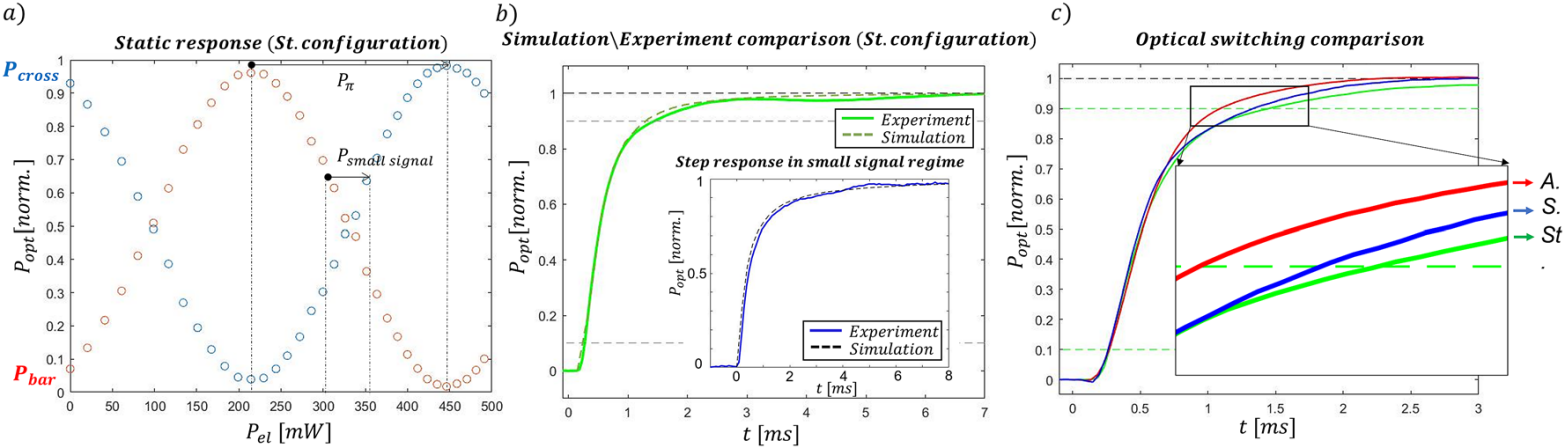
(**a**) Static response of the MZI at increasing values of dissipated power (standard configuration). (**b**) comparison between simulated and experimental switching time and, in the inset, temporal response comparison to a step like applied power in the small signal regime (standard configuration). (**c**) Switching time comparison between the three different configurations: asymmetric (A.), symmetric (S.) and standard (St.).

To validate our simulations, we have fabricated three different integrated optical switches, implementing the *standard*, the *symmetric* and the *asymmetric* configurations. We have started investigating the dynamics of the standard configuration. To do this measurement, we have first characterized the static response of the corresponding MZI at different values of dissipated power, collecting the output light distribution from bar and cross. The results of this measurement are illustrated in Fig. 5a. This allowed the identification of the required power and voltage range to perform the entire optical switch (P_π_), as well as the values that permit to work in the linear regime (P_small signal_). It is worth noticing that the measured P_π_ is higher than the simulated one, about 230 mW, instead of 197 mW. We attribute this discrepancy to the ubiquitous gold deposition on the glass surface that could affect the heat diffusion. We have subsequently compared the simulated and measured dynamics of the device in the standard configuration, as illustrated in Fig. 5b. The experimental values have been averaged over four different acquisitions. Both the optical switch as well as the step response in the small signal regime (illustrated in the inset) show a good overlap between the two dynamics, highlighting the reliability of the simulations. We have subsequently compared the switching time of the three configurations (*standard, symmetric* and *asymmetric*). In this case, the switching time is defined as the time difference between the 90% and 10% values of the response (t_switch_ = t_90% _− t_10%_) and represents the standard way to assess the speed of a switch. First, we have retrieved the static response of the other two devices to identify the correct power range for the optical switch. P_π_ corresponds to around 155 mW for both the *symmetric* and *asymmetric* configurations. Despite not having measured any difference in power dissipation among these two layouts, we have confirmed that the presence of trenches reduces the necessary power dissipation to perform the switch with respect to the standard configuration. The result of this comparison is shown in Fig. 5c where the three dynamics are reported, and the expected performances are confirmed. As shown, the asymmetric configuration is the fastest one with a switching time of about 830 μs, while the symmetric and the standard ones present a measured switching time of about 1 ms and 1.18 ms, respectively. It is worth highlighting that the measured switching times are, as expected, different from τ, due to the non-linear response of the MZI (see Eqs. 1, 2, 4)^24^.

### Switch based on an optimized voltage function

To further speed up the system we have investigated a pulse shaping approach, thus we have decided to optimize the applied power function to obtain a step-like response of the system. In particular, instead of applying a step-like power, the new function consists in a first peak step, which promptly provides all the energy needed by the system to reach the required temperature difference, followed by a second step at the regime power, which permits to maintain the required shift. Indeed, if we consider linear system theory, given a certain input function x(t), the output function y(t) is the convolution between x(t) and the system transfer function h(t). If we now take into account the corresponding Laplace transform, we obtain the following product:

5 $$\begin{array}{*{20}c} {Y\left( s \right) = X\left( s \right)H\left( s \right) } \\ \end{array}$$

If we approximate our system to a single pole one, its transfer function corresponds to:

6 $$\begin{array}{*{20}c} {H\left( s \right) = \frac{k}{1 + s\tau } } \\ \end{array}$$

where k is the transfer function value at zero frequency and τ is the time constant of the system. Considering that we aim to obtain a step like response, the desired output function corresponds to:

7 $$\begin{array}{*{20}c} {Y\left( s \right) = \frac{1}{s}} \\ \end{array}$$

Therefore, we can retrieve the corresponding input function in the following way:

8 $$\begin{array}{*{20}c} {X\left( s \right) = \frac{Y\left( s \right)}{{H\left( s \right)}} = \frac{1}{sk} + \frac{\tau }{k} } \\ \end{array}$$

This corresponds to a time dependent input which is the sum of an initial delta δ(t), with an area τ/k, and a second step (Heaviside function He(t)), with an amplitude 1/k:

9 $$\begin{array}{*{20}c} {x\left( t \right) = \frac{1}{k}He\left( t \right) + \frac{\tau }{k}\delta \left( t \right) } \\ \end{array}$$

**Figure 6:**
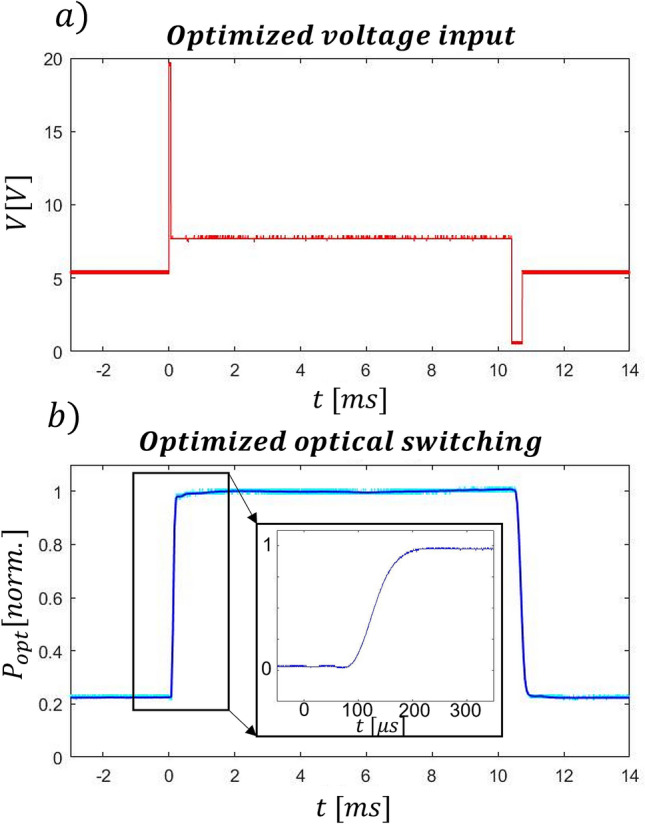
(**a**) Optimized voltage function to achieve the fastest output switching. (**b**) Corresponding output power, with a τ_ON_ and τ_OFF_ equal to 78 μs and 209 μs, respectively (asymmetric configuration).

A good approximation can be obtained by substituting the delta function with a short, squared voltage peak that has a total area (i.e. energy) equal to the area of the delta function. In our case, we were limited by the maximum voltage of the function generator, which is equal to 20 V. Using the asymmetric switch configuration, we have retrieved the corresponding peak duration experimentally, as the time range that allows the system to reach the phase shift necessary to switch between bar and cross. The optimal peak power duration was as short as 65 µs. In this configuration we have been able to demonstrate a rapid switch on–off and vice versa, as illustrated in Fig. 6. The measured time to switch on the system is equal to 78 μs (τ_ON_), while the one to switch it off is about 209 μs (τ_OFF_). This difference in value is due to the unbalanced available power peak that can be applied in the rising and lowering processes, which in turn depends on the power values required to switch between bar and cross (P_π_), as measured through the static response analysis (Fig. 5.a). Regarding the overall power consumption, the peak power applied to the system in Fig. 6 is around 2.3 W maintained for 65 µs, which is then lowered to 392 mW to keep the phase shift constant. It is worth noting that, notwithstanding the high peak power in the first pulse, the dissipated energy for the switching operation is ideally equal to that dissipated over a longer time interval with the single step actuation. The thermal budget that needs to be managed on the chip is therefore the same.

## Conclusions

In this work, we have investigated the dynamic response of thermal phase shifters in glass photonic circuits. We have first optimized the device layout in order to speed up the system. Taking advantage of the versatility of FLM, we have introduced features that alter the static and dynamic response of the system, such as superficial trenches and conductive layers. We have then applied two different configurations to a MZI based thermo-optical switch, demonstrating a device that is capable to commute the output state in 0.83 ms with only 155 mW of dissipated electrical power. This result is about an order of magnitude faster than other glass-based thermo-optical switches, previously presented in the literature^16–20^. In addition, by implementing a voltage pulse shaping approach, we have been able to demonstrate an additional dramatic improvement in the system response, with a switching time of about 78 μs. We believe this result will allow increasing the range of applications of reconfigurable photonic circuits based on thermal phase shifters, which were previously limited by the slow temporal response. For instance, the straightforward fabrication of both optical and microfluidic components in glass substrates by FLM will permit to obtain lab-on-a-chip devices for a rapid and reconfigurable sample analysis. 

## Data Availability

All data needed to support the conclusions of the paper are present in the paper. The unprocessed data may be requested to the authors.
